# Inhibition Effects and Mechanisms of Marine Compound Mycophenolic Acid Methyl Ester against Influenza A Virus

**DOI:** 10.3390/md22050190

**Published:** 2024-04-23

**Authors:** Zihan Wang, Lishan Sun, Hongwei Zhao, Mamadou Dioulde Sow, Yang Zhang, Wei Wang

**Affiliations:** Key Laboratory of Marine Drugs of Ministry of Education, Shandong Provincial Key Laboratory of Glycoscience and Glycoengineering, School of Medicine and Pharmacy, Ocean University of China, 5 Yushan Road, Qingdao 266003, China; wzzz0911@163.com (Z.W.); 17806274691@163.com (L.S.); hweizhao2024@163.com (H.Z.); sowm0055@gmail.com (M.D.S.); zhangyang993@163.com (Y.Z.)

**Keywords:** influenza A virus, MAE, anti-viral effect, Akt-mTOR-S6K pathway, viral pneumonia

## Abstract

Influenza A virus (IAV) can cause infection and illness in a wide range of animals, including humans, poultry, and swine, and cause annual epidemics, resulting in thousands of deaths and millions of hospitalizations all over the world. Thus, there is an urgent need to develop novel anti-IAV drugs with high efficiency and low toxicity. In this study, the anti-IAV activity of a marine-derived compound mycophenolic acid methyl ester (MAE) was intensively investigated both in vitro and in vivo. The results showed that MAE inhibited the replication of different influenza A virus strains in vitro with low cytotoxicity. MAE can mainly block some steps of IAV infection post adsorption. MAE may also inhibit viral replication through activating the cellular Akt-mTOR-S6K pathway. Importantly, oral treatment of MAE can significantly ameliorate pneumonia symptoms and reduce pulmonary viral titers, as well as improving the survival rate of mice, and this was superior to the effect of oseltamivir. In summary, the marine compound MAE possesses anti-IAV effects both in vitro and in vivo, which merits further studies for its development into a novel anti-IAV drug in the future.

## 1. Introduction

Influenza A virus (IAV) is a formidable pathogen which can cause contagious respiratory disease with potential fatality in humans, poultry, and swine [1]. Official WHO (World Health Organization) statistics indicate that the seasonal flu caused by H1N1 and H3N2 virus can cause serious infections in 3–5 million individuals, resulting in about 290,000–650,000 deaths per year worldwide [2,3]. However, only three classes of anti-IAV drugs have been approved for marketing: the ion-channel blockers amantadine and rimantadine [4], the neuraminidase inhibitors zanamivir, oseltamivir, and paramivir [5], and the PA protein inhibitor baloxavir [6]. Despite these successes, drug resistance and toxicity are still unresolved issues in the fight against IAV infection [7]. Thus, the development of novel anti-IAV drugs with high efficiency and low toxicity is urgently needed.

Marine organisms have produced a large number of structurally novel marine active molecules including algae polysaccharides, marine peptides, and microbial secondary metabolites [8]. Mycophenolic acid (MPA) is a non-competitive and reversible inhibitor of dehydrogenase inosine-5′-monophosphate (IMPDH) and possesses broad pharmacological activities such as antibacterial, antifungal, antiviral, immunosuppressive, and anticancer properties [9]. The antiviral activity of mycophenolic acid and its derivatives has been widely reported; as early as 2007, Johan Neyts’ team [10] found that mycophenolic acid (MPA) and its derivative mycophenolate mofetil (MMF) significantly enhanced the anti-HBV activity of guanine nucleoside analogues. Subsequently, Kevin Chiem et al. [11] found that MPA significantly inhibited Monkeypox virus (MPXV) replication in vitro. In addition, MPA can inhibit viral RNA replication by depleting the intracellular guanosine pool [12] and shows strong anti-influenza virus activities [13].

Through activity screening, we discovered a MPA derivative mycophenolic acid methyl ester (MAE) isolated from marine fungus *Phaeosphaeria spartinae* had marked anti-IAV effects in vitro, thus the anti-IAV effects and mechanisms of MAE were investigated both in vitro and in vivo in this study. The results showed that MAE may be able to activate cellular Akt-mTOR-S6K pathway so as to inhibit viral replication. Importantly, oral administration of MAE can protect mice against IAV-induced death and weight loss, superior to the effects of the clinical drug oseltamivir, suggesting that MAE has potential to be developed into a novel anti-IAV agent in the future.

## 2. Results

### 2.1. Marine Compound MAE Suppresses IAV Multiplication In Vitro with Low Toxicity

**Figure 1:**
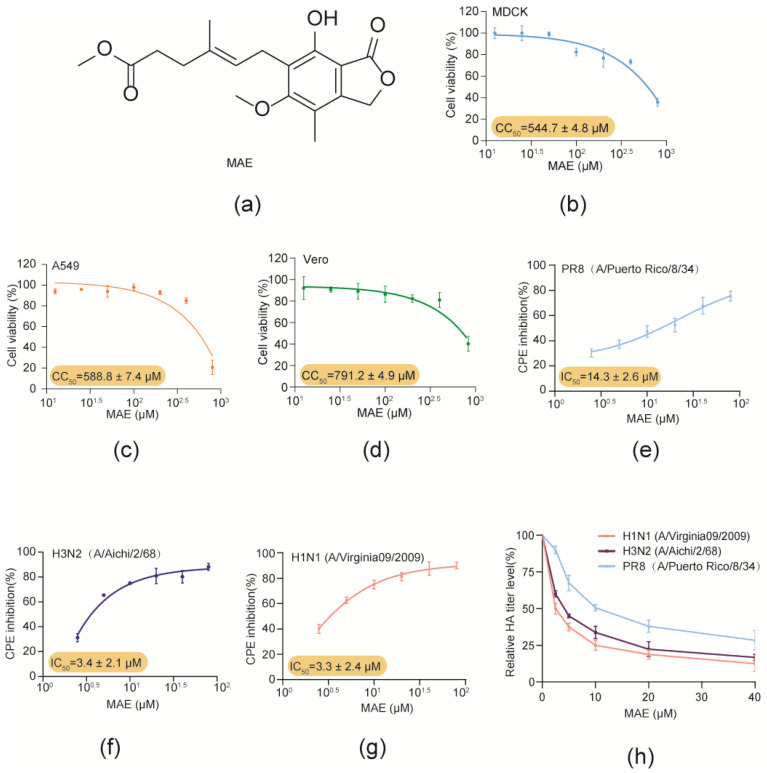
Inhibition effects of MAE against IAV infection in vitro. (**a**). Structure of MAE. (**b**–**d**) The cytotoxicity of MAE in MDCK, Vero and A549 cells. Values are means ± S.D. (n = 3). (**e**–**g**) Anti-IAV (PR8, Aichi, Virginia09; MOI = 0.1) activity of MAE (2.5–80 μM) was determined by CPE inhibition assay at 24 h p.i. Values are means ± S.D. (n = 3). (**h**) The inhibition of different concentrations of MAE (2.5–40 μM) on IAV multiplication was evaluated by HA assay. Values are means ± S.D. (n = 3).

In this study, the anti-IAV effects and mechanisms of marine-derived compound MAE (Figure 1a) were investigated both in vitro and in vivo. Firstly, the cytotoxicity of MAE in MDCK, Vero and A549 cells was determined by MTT assay. The results showed that MAE exhibited no significant cytotoxicity at concentrations ranging from 12.5 to 800 μM (Figure 1b–d). The CC_50_ (50% cytotoxicity concentration) values of MAE in MDCK, A549 and Vero cells were about 544.7 ± 4.8 μM, 588.8 ± 7.4 μM, and 791.2 ± 4.9 μM, respectively. These results were used to determine the dose range of MAE for the subsequent experiments.

Then the anti-IAV activity of MAE was evaluated by CPE inhibition assay and hemagglutination (HA) titer assay in PR8, Aichi or Vir09 virus-infected MDCK cells [14,15,16]. As shown in Figure 1e–g, MAE (2.5–80 μM) treatment dose-dependently promoted the viability of IAV-infected cells, and the IC_50_ values obtained for MAE inhibition of PR8, Aichi and Vir09 virus were about 14.3 ± 2.6 μM, 3.4 ± 2.1 μM, and 3.3 ± 2.4 μM, respectively. In addition, MAE also significantly reduced the PR8, Aichi and Vir09 virus HA titers in a concentration-dependent manner with the IC_50_ value of 13.31 ± 2.2 μM, 4.03 ± 1.3 μM, and 2.9 ± 1.2 μM. Thus, MAE possesses anti-IAV activity in vitro with high efficiency and low toxicity.

### 2.2. MAE Inhibits Both the Expression of mRNA and Protein of IAV

**Figure 2:**
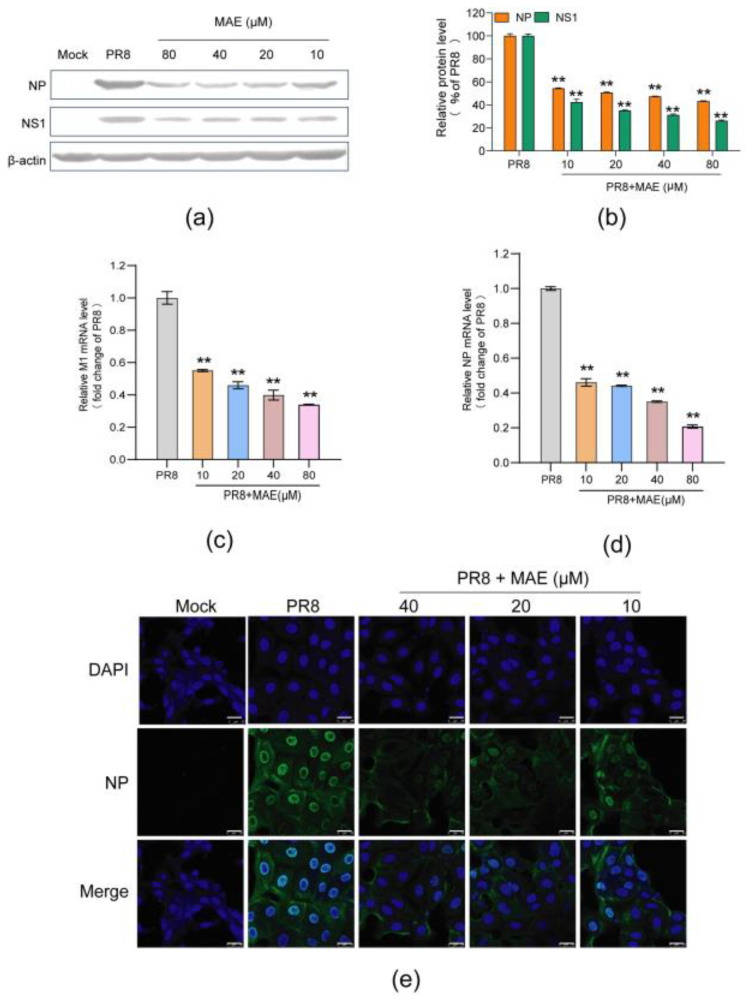
Inhibition of MAE on the protein and mRNA expression of IAV. (**a**,**b**) The inhibition of MAE on IAV multiplication was also evaluated by Western blot assay of virus NP and NS1 proteins in MDCK cells (**a**). Quantification of immunoblot for the ratio of NP or NS1 protein to β-actin was also shown (**b**). Values are means ± S.D. (n = 3). ** *p* < 0.01 vs. virus control group (PR8). (**c**,**d**) Quantitative RT-PCR assay of virus M1 (**c**) or NP (**d**) mRNA in MAE-treated cells was performed. Values are means ± S.D. (n = 3). ** *p* < 0.01 vs. virus control group (PR8). (**e**) PR8 (MOI = 1.0)-infected MDCK cells were treated with or without MAE after virus adsorption and then incubated at 37 °C for 4 h. After that, NP protein expression was determined by immunofluorescence assay. Scale bar represents 25 μm.

To further investigate the in vitro anti-IAV activity of MAE, Western blot assay was performed to evaluate the influence of MAE on virus protein expression in IAV-infected cells [17]. As shown in Figure 2a,b, MAE (10–80 μM) treatment significantly reduced the expression of viral NP and NS1 proteins. Besides that, the results of quantitative RT-PCR assay showed that MAE treatment also significantly reduced the mRNA levels of virus NP and M1 genes in a dose-dependent manner (Figure 2c,d), suggesting that MAE could effectively inhibit both the mRNA and protein expression of IAV.

Moreover, the inhibitory effect of MAE on the infection of IAV was further evaluated by indirect immunofluorescence assay [18]. As shown in Figure 2e, the results showed that MAE (10–40 μM) treatment significantly reduced the green fluorescence of virus NP protein in both cytoplasm and nucleus, suggesting that MAE can truly inhibit the expression of NP protein in IAV-infected cells. Taken together, MAE possesses marked inhibition on IAV multiplication in vitro.

### 2.3. Influence of Different Treatment Conditions of MAE on IAV Infection

**Figure 3:**
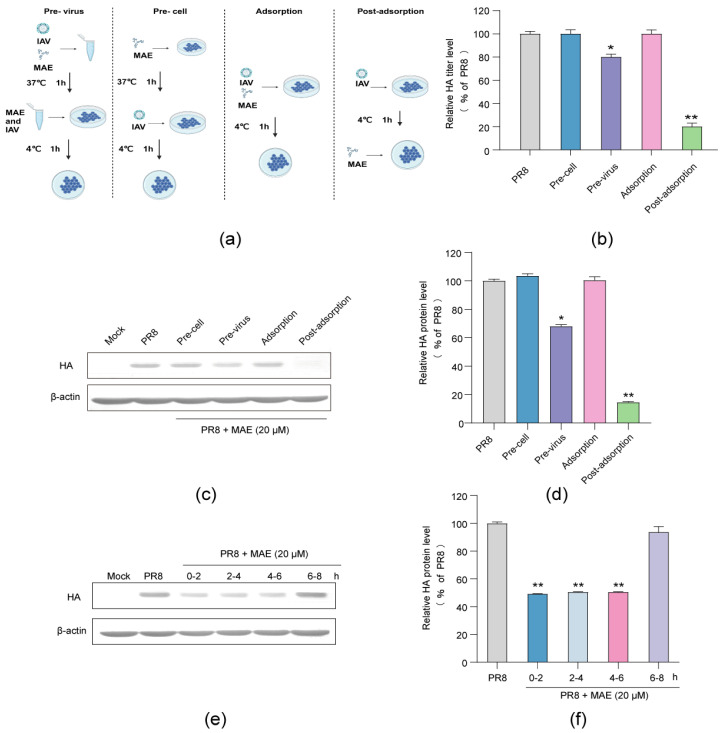
Influence of different treatment conditions of MAE on IAV infection. (**a**) The schematic diagram of different treatment conditions. (**b**) MDCK cells were infected with PR8 virus (MOI = 1.0) under four treatment conditions of MAE (20 μM) and the antiviral activity was determined by HA assay at 24 h p.i. Values are means ± S.D. (n = 3). * *p* < 0.05, ** *p* < 0.01 vs. PR8 group. (**c**,**d**) Effects of the MAE on HA protein expression under different treatment conditions. Quantification of immunoblot for the ratio of HA to β-actin was also shown (**d**). Values are means ± S.D. (n = 3). * *p* < 0.05, ** *p* < 0.01 vs. PR8 group. (**e**,**f**) PR8 (MOI = 1.0)-infected MDCK cells were treated with MAE (20 μM) for different time intervals, after which (at 8 h p.i.) the virus yields were determined via Western blot assay of virus HA protein (**e**). Quantification of immunoblot for the ratio of HA to β-actin was also shown (**f**). Values are means ± S.D. (n = 3). ** *p* < 0.01 vs. virus control group (PR8).

To further investigate the stage(s) at which MAE exerts its inhibitory effect, the time-of-addition assay of MAE was performed under four different conditions (Figure 3a) [19,20]. The results of HA titer showed that pretreatment of PR8 virus (MOI = 1.0) with MAE (20 μM) for 1 h prior to infection significantly inhibited viral multiplication in MDCK cells, suggesting that MAE may have a direct interaction with PR8 virus to some extent (Figure 3b). Treatment with MAE post adsorption showed a more significant inhibitory effect on the viral HA titer, suggesting that MAE may exert its anti-IAV actions mainly through blocking some steps after virus adsorption (Figure 3b). Similarly, the results of Western blot assay also indicated that pretreatment of virus with MAE or treatment of MAE post adsorption can significantly reduce the expression levels of virus HA protein in IAV-infected cells (Figure 3c,d).

Furthermore, another time course study within 8 h was also performed to further determine the specific viral stage post-adsorption inhibited by MAE. As shown in Figure 3e,f, the results of Western blot assay showed that treatment with MAE (20 μM) during the first six hours after adsorption (0–6 h p.i.) significantly reduced the production of HA protein to about 50% of the virus control group (*p* < 0.01). However, no significant inhibition on HA production was noted when MAE was added later than 6 h post-infection (6–8 h p.i.) (Figure 3e,f). Thus, MAE may mainly inhibit the early and intermediate stages (0–6 h p.i.) of the IAV life cycle after adsorption.

### 2.4. MAE May Inhibit IAV Infection through Activating Cellular Akt-mTOR-S6K Pathway

**Figure 4:**
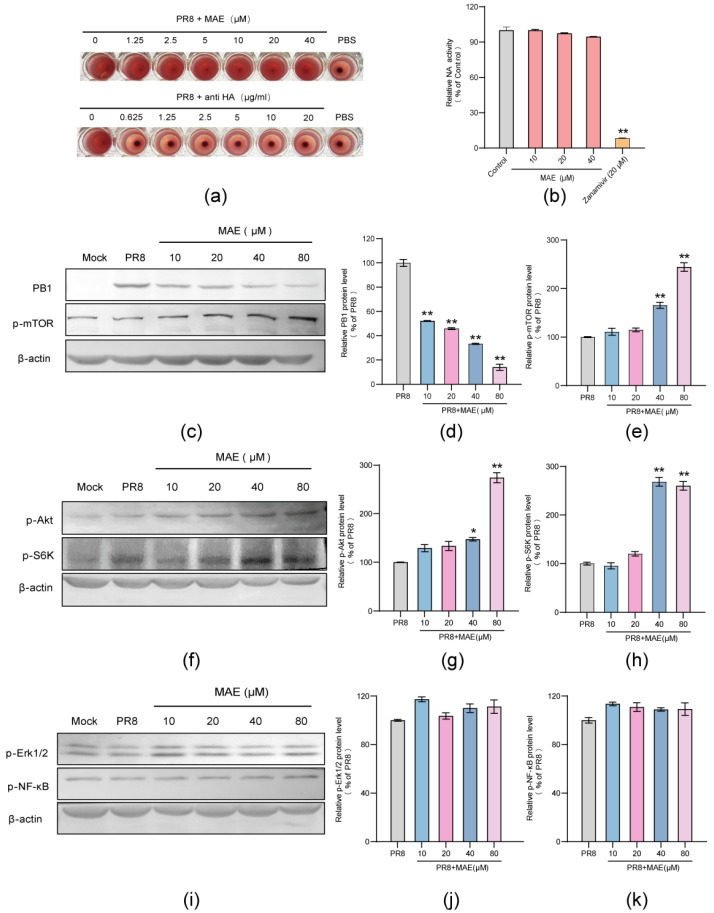
Enhancement of Akt-mTOR-S6K pathway by MAE in IAV-infected cells. (**a**) The inhibition effects of MAE and anti-HA antibody on PR8 virus-induced aggregation of chicken erythrocytes were evaluated by hemagglutination inhibition (HI) assay. (**b**) Inactivated PR8 virus was incubated with indicated concentrations of MAE or Zanamivir (20 μM), and the NA activity was determined by a fluorescent assay. Values are means ± S.D. (n = 3). ** *p* < 0.01 vs. Control group. (**c**) PR8 (MOI = 1.0) infected A549 cells were treated with or without MAE at indicated concentrations after removal of virus inoculums. At 5 h p.i., PB1 and p-mTOR protein were determined by Western blotting. (**d**,**e**) Quantification of immunoblot for the ratio of PB1 or p-mTOR to β-actin. Values are means ± S.D. (n = 3). ** *p* < 0.01 vs. PR8 group. (**f**) PR8 (MOI = 1.0) infected A549 cells were treated with or without MAE at indicated concentrations after removal of virus inoculums. At 5 h p.i., p-Akt and p-S6K protein were determined by Western blotting. (**g**,**h**) Quantification of immunoblot for the ratio of p-Akt or p-S6K to β-actin. Values are means ± S.D. (n = 3). * *p* < 0.05, ** *p* < 0.01 vs. PR8 group. (**i**–**k**) PR8 (MOI = 1.0) infected A549 cells were treated with or without MAE after removal of virus inoculums. At 5 h p.i., p-ERK1/2 and p-NF-κB protein were determined by Western blotting (**i**). Quantification of immunoblot for the ratio of p-ERK1/2 and p-NF-κB to β-actin (**j**,**k**). Values are means ± S.D. (n = 3).

Since MAE may interact with virus particles or inhibit some steps after virus adsorption, we first investigate whether MAE affects the functions of surface protein HA and NA of IAV by using the HA inhibition and NA inhibition assay. As shown in Figure 4a, the results showed that the anti-HA antibodies significantly inhibited the PR8 virus-induced aggregation of chicken erythrocytes at the concentrations of 0.625–20 μg/mL, while MAE could not inhibit aggregation of chicken erythrocytes even at a concentration of 40 μM, suggesting that MAE may have no direct interaction with viral HA protein.

To further determine whether MAE can bind to NA protein to block the release of viral particles, the NA inhibition assay was performed with NA inhibitor Zanamivir as the positive control. The results showed that MAE (10–40 μM) nearly had no inhibition effect on the activity of NA protein, while Zanamivir inhibited about 95% of NA activity at 20 μM (Figure 4b), suggesting that virus NA protein may be not the target of MAE.

Since MAE may largely inhibit some stages of IAV life cycle after adsorption, the influence of MAE on host signaling pathways associated with IAV infection was further explored. The Akt-mTOR-S6K pathway has been reported to be critical for TLR mediated induction of type I interferon, and the activation of S6K, a signaling molecule downstream of mTOR, promotes the production of type I interferon, which plays a crucial role in anti-IAV immunity [21,22,23]. Thus, we further explored the effect of MAE on the activation of mTOR, Akt and S6K by Western blot assay. The results showed that treatment with MAE (40, 80 μM) for 5 h after adsorption significantly increased the expression levels of p-mTOR, p-Akt and p-S6K to about 1.5–2.5 fold of the virus control group (PR8) (*p* < 0.05), respectively (Figure 4c–h). The expression level of viral protein PB1 also decreased significantly in a dose-dependent manner after treatment of MAE post adsorption (Figure 4d). Thus, the Akt-mTOR-S6K pathway may be involved in the anti-IAV actions of MAE in vitro. In addition, MAE (10–80 μM) treatment had no significant influence on the activation of ERK1/2 and NF-kB which are associated with IAV infection (*p* > 0.05) (Figure 4i–k), suggesting that MAPK and NF-kB pathway may not be the anti-IAV targets of MAE. Taken together, the cellular Akt-mTOR-S6K pathway can be enhanced by MAE treatment in IAV-infected cells, and the activation of S6K may promote the production of type I interferon, which in turn leads to the inhibition of IAV replication.

### 2.5. Oral Efficacy of MAE against IAV Infection In Vivo

**Figure 5:**
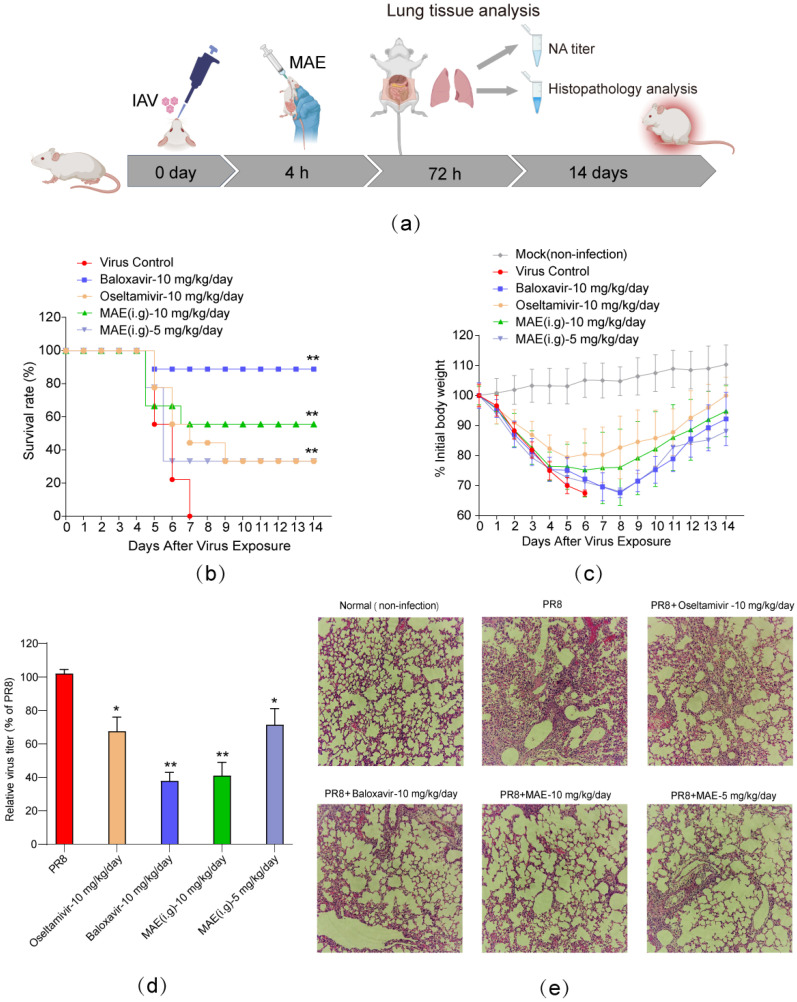
The anti-IAV activities of MAE in vivo. (**a**) Schematic diagram of IAV mouse pneumonia model. (**b**) Survival rate. IAV-infected mice received oral therapy with MAE or other drugs for five days. Results are expressed as the percentage of survival, evaluated daily for 14 days. (**c**) The average body weights were monitored daily for 14 days and are expressed as a percentage of the initial value. Values are means ± S.D. (n = 10). (**d**) The pulmonary viral titers were evaluated by NA titer assay. Values are means ± S.D. (n = 4). * *p* < 0.05, ** *p* < 0.01 vs. PR8 group. (**e**) Histopathologic analyses of lung tissues on day 3 p.i. by hematoxylin-eosin (H&E) staining (×100).

The anti-IAV effects of MAE in vivo were then tested in a murine pneumonia model as previously described [24] (Figure 5a). Briefly, PR8 virus-infected mice received oral administrations of MAE (5 or 10 mg/kg/day), oseltamivir (10 mg/kg/day), baloxavir (10 mg/kg/day), or PBS (virus control) once a day for five days, and then the survival of mice was evaluated daily for 14 days. The results showed that oral administration of MAE (10 mg/kg/day) significantly increased the survival rate to about 60% on day 14, superior to the effect of oseltamivir (10 mg/kg/day; 40%). It should also be noted that the positive drug baloxavir (10 mg/kg/day; 90%) is superior to the compound MAE (Figure 5b). In addition, the mice in the virus control group continued to lose weight before they all died on day 7 (Figure 5c). However, the MAE (10 mg/kg/day)-treated mice only decreased their body weights within the first four days before gradually regaining their body weights to the initial levels, comparable to the effect of oseltamivir (10 mg/kg/day).

To further evaluate the inhibitory effect of MAE on IAV infection in vivo, the viral titer in the lungs of mice was determined by the NA titer assay. As shown in Figure 5d, oral administration of MAE (10 mg/kg/day) significantly decreased the pulmonary viral titers in the mouse pneumonia model as compared to the virus control group (*p* < 0.01), comparable to the effect of baloxavir (10 mg/kg/day). MAE (5 mg/kg/day) treatment also significantly reduced virus pulmonary titers (*p* < 0.05), similar to the effect of oseltamivir. Moreover, the histopathological analysis also showed that after oral treatment of MAE (5 or 10 mg/kg/day) for 3 days, the IAV-infected mice had intact columnar epithelia in the bronchiole without inflammatory cell infiltration, comparable to the effect of oseltamivir (10 mg/kg/day) and baloxavir (10 mg/kg/day) (Figure 5e). Thus, MAE also possessed anti-IAV activities in vivo.

## 3. Discussion

Recently, natural products derived from marine microorganisms have attracted increasing interest for the development of potential antiviral drugs [25,26]. In the present study, a marine-derived compound, MAE, was found to be able to suppress the infection of different IAV strains in vitro with low toxicity. MAE may block virus replication via promoting the activation of the intracellular Akt-mTOR-S6K pathway. Importantly, oral administration of MAE significantly decreased the pulmonary viral titers and improved the survival rate in IAV-infected mice, suggesting that MAE has the potential to be developed into a novel anti-IAV agent.

Virus–host interactions are complex, and viruses not only need to exploit a number of host signaling pathways for their own life cycle, but must also evade or counteract signaling pathways for inflammatory, innate, and acquired immune responses activated by viral infection [27]. The intracellular Akt-mTOR-S6K pathway has been reported to regulate a variety of physiological activities, including cell proliferation, growth, metabolism, autophagy, angiogenesis, and metastasis [28]. The Akt-mTOR-S6K pathway also plays a crucial role during viral invasion of cells. Besides that, viral infection can activate the Akt-mTOR pathway, which can lead to cellular autophagy [29], and on the other hand the activation of cellular Akt-mTOR-S6K pathway, especially the downstream signaling S6K, has been reported to promote the secretion of type I interferon [21,23], which plays a crucial role in the innate immune antiviral process [30].

Herein, the time-of-addition assay indicated that MAE may largely inhibit some stages (0–6 h p.i.) of IAV life cycle after adsorption, suggesting that MAE may directly promote the activation of intracellular signaling pathways to interfere with the entry or replication of IAV. However, MAE had no inhibition on the function of HA and NA protein of IAV, suggesting that MAE did not inhibit virus entry process. Interestingly, MAE can significantly enhance the activation of Akt-mTOR-S6K signaling pathway rather than MAPK and NF-κB pathways. Thus, MAE treatment may promote the activation of S6K in IAV-infected cells so as to enhance the production of type I interferon, which in turn leads to the inhibition of IAV replication.

The PR8 virus-induced murine pneumonia model has been established and used for studying the anti-IAV effects of MAE in vivo [24]. Herein, we found that oral therapy of MAE significantly reduced the pulmonary viral titers, and improved the survival rate of mice, superior to the effect of oseltamivir. MAE treatment also attenuated the weight loss of mice and alleviated the pneumonia symptoms in IAV-infected lungs, comparable to the effects of oseltamivir and baloxavir, suggesting that MAE also possessed marked anti-IAV activities in vivo. Taken together, our studies indicate that gavage administration of MAE had remarkable anti-IAV effects, which suggested that MAE may be used for treatment of influenza disease by oral therapy in the future.

## 4. Materials and Methods

### 4.1. Reagents, Cells, and Viruses

Mycophenolic acid methyl ester has been isolated from an EtOAc extract of *Phaeosphaeria spartinae*, which is an endophyte isolated from the marine alga *Ceramium* sp. Fractionation of the EtOAc extract of a solid culture of *Phaeosphaeria spartinae* involving VLC and HPLC furnished the compounds MAE [31]. MAE (with purity > 99%) was purchased from Topscience (Shanghai, China). MDCK cells were grown in RPM1640 medium (Thermo Fisher Scientific, Waltham, MA, USA) supplemented with 10% fetal bovine serum (FBS) (ExCell Bio, Shanghai, China), 100 U/mL of penicillin, and 100 μg/mL of streptomycin (NCM Biotech, Suzhou, China). Vero cells were maintained in Modified Eagle’s medium (MEM) (Thermo Fisher Scientific, Waltham, MA, USA) containing 10% FBS, 100 U/mL of penicillin, and 100 μg/mL of streptomycin. A549 cells were grown in Ham’s F-12K medium (Thermo Fisher Scientific, Waltham, MA, USA) supplemented with 10% FBS, 100 U/mL of penicillin, and 100 μg/mL of streptomycin. Influenza virus (A/Puerto Rico/8/34 [H1N1]; PR/8) was propagated in 9-day-old embryonated eggs for 3 days at 36.5 °C. Influenza H1N1 virus A/Virginia/ATCC1/2009 (Vir09) and H3N2 strain A/Aichi/2/1968 (Aichi) were propagated in MDCK cells at 37 °C for 3 days. The virus titers were determined by hemagglutination assay and neuraminidase assay [32].

### 4.2. Cytotoxicity Assay

The cytotoxicity of compounds was determined by the MTT (3-(4,5-dimethylthiazol-2-yl)-2,5-diphenyltetrazolium bromide; (Sigma-Aldrich, St. Louis, MO, USA) assay. Briefly, confluent MDCK, A549, Vero cell cultures in 96-well plates, were exposed to different concentrations of MAE (12.5, 25, 50, 100, 200, 400, 800 μM) in triplicate for 48 h. Next, 10 μL of PBS containing MTT (final concentration: 0.5 mg/mL) was added to each well. After incubation for 4 h at 37 °C, the supernatant was removed, and 200 μL of DMSO was added to each well to dissolve the formazan crystals. After vigorous shaking, absorbance values were measured at 570 nm using a microplate reader (Bio-Rad, Hercules, CA, USA). CC_50_ was calculated as the concentration of compound required to reduce cell viability by 50%.

### 4.3. Cytopathic Effect (CPE) Inhibition Assay

The antiviral activity was evaluated by the CPE inhibition assay as described previously [10]. In brief, MDCK cells in 96-well plates were firstly infected with IAV (MOI = 0.1), and then treated with different compounds in triplicate after removal of the virus inoculum. After 48 h incubation, the cells were fixed with 4% formaldehyde (Leagene, Shanghai, China) for 20 min at room temperature (RT). After removal of the formaldehyde, the cells were stained with 0.1% crystal violet for 30 min. The plates were washed and dried, and the intensity of crystal violet staining for each well was measured at 570 nm. The concentration required for a test compound to reduce the CPE of IAV by 50% (IC_50_) was determined.

### 4.4. Indirect Immunofluorescence Assay

PR8 virus (MOI = 1.0) infected MDCK cells were treated with or without MAE (10–40 µM) after adsorption. After four hours incubation at 37 °C, the cells were fixed, permeabilized, and incubated sequentially with anti-IAV NP protein primary antibody (12 h) and fluorescein isothiocyanate (FITC)-conjugated secondary antibody (1 h) (Boster, Wuhan, China) at 4 °C. Then, the cell nucleus was stained with DAPI (Beyotime, Shanghai, China) for 20 min before confocal imaging. Finally, cells were washed and directly observed using Laser Scanning Confocal Microscope (Leica DMI6000B, Wetzlar, Germany).

### 4.5. Real-Time RT-PCR Assay

PR8 (MOI = 1.0) infected A549 cells were treated with different concentrations of compounds after virus adsorption. Then the total RNA was extracted at 6 h p.i. using an RNAiso™Plus Kit (Takara, Japan), and analyzed by using the One Step SYBR PrimeScript RT-PCR Kit (Takara, Koufushi, Japan). The primer pairs for IAV NP, M1 and cellular β-actin mRNA were listed as follows: NP mRNA, 5′-ACGGTACCATGGCGTCTCAA-3′ and 5′-TCACTCTAGATCAATTGTCATA-3′; M1 mRNA, 5′AAACATATGTCTGATAACGAAGGAGAACAGTTCTT-3′ and 5′GCTGAATTCTACCTCATGGTCTTCTTGA-3′; human β-actin mRNA, 5′-AACAGTCCGCCTAGAAGCAC-3′ and 5′- CGTTGACATCCGTAAAGACC-3′. The real-time RT-PCR was performed at 42 °C 5 min, 95 °C 10 s, 40 cycles of 95 °C 5 s, 60 °C 34 s, followed by melting curve analysis, according to the instrument documentation (ABI PRISM 7500, Applied Biosystems, Carlsbad, CA, USA). All reactions were performed in triplicate and the results were normalized to β-actin. The relative amounts of IAV NP and M1 mRNA molecules were determined using the comparative (2^−ΔΔCT^) method, as previously described [33].

### 4.6. Time-of-Addition Assay

MDCK cells were infected with PR8 virus (MOI = 1.0) under four different treatment conditions: (i) Pretreatment of virus: MAE (20 μM) pretreated IAV was added to MDCK cells and incubated at 37 °C for 1 h. Then after adsorption, the virus inoculum containing MAE was removed and the cells were overlaid with compound-free media. (ii) Pretreatment of cells: MDCK cells were pretreated with 20 μM of MAE before IAV infection. (iii) Adsorption: MDCK cells were infected in media containing MAE (20 μM) at 4 °C for 1 h. After that, the virus inoculum was removed and the compound-free media were added into cells. (iv) Post-adsorption: after removal of unabsorbed virus, MAE (20 μM) was added to the cells. At 8 h p.i., virus yields were determined by HA assay.

Moreover, another time course study was also performed to explore which viral stage after adsorption is inhibited by MAE. Briefly, PR8 virus (MOI = 1.0) infected MDCK cells were treated with 20 μM of MAE for different time intervals (0–2 h p.i., 2–4 h p.i., 4–6 h p.i., 6–8 h p.i.), after which (at 8 h p.i.) the virus yields were determined via Western blot assay of virus HA protein. The relative densities of protein bands were determined by Image J (NIH) V.1.33 u (USA).

### 4.7. Hemagglutination (HA) Assay

Standardized chicken red blood cell (cRBC) solutions were prepared according to the WHO manual. Virus propagation solutions were serially diluted 2-fold in round bottomed 96-well plate and 1% cRBCs were then added at an equal volume. After 60 min incubation at 4 °C, RBCs in negative wells sedimented and formed red buttons, whereas positive wells had an opaque appearance with no sedimentation. HA titers are given as hemagglutination units/mL (HAU/mL).

### 4.8. Neuraminidase Inhibition Assay

The influenza neuraminidase inhibitor detection kit (Beyotime, Shanghai, China) was used to measure the inhibition of NA activity as described previously [32]. Briefly, inactivated PR8 virus supernatants were added to a 96-well plate and then mixed with MAE at different concentrations (10, 20, 40 μM) or zanamivir (20 μM) (diluted in 33 mM MES buffer (pH 3.5), 4 mM CaCl_2_) at 37 °C for 30 min. Then MUNANA (20 μM) (Sigma-Aldrich, St. Louis, MO, USA) was added as the substrate and incubated at 37 °C for 40 min. The reaction was terminated by the addition of stop solution (25% ethanol, 0.1 M glycine, pH 10.7). Fluorescence was measured using a SpectraMax M5 plate reader (Sunnyvale, CA, USA) with excitation and emission wavelengths of 360 and 440 nm, respectively.

### 4.9. Western Blot Assay

After drug treatment, the cell lysate was separated by SDS-PAGE and transferred to nitrocellulose membrane. After being blocked in Tris-buffered saline (TBS) containing 0.1% Tween 20 (*v*/*v*) and 5% BSA (*w*/*v*) at RT for 2 h, the membranes were rinsed and incubated at 4 °C overnight with primary antibodies against virus NP, NS1, HA proteins (Santa Cruz Biotechnology, Santa Cruz, CA, USA) or β-actin antibodies (Cell Signaling Technology, Danvers, MA, USA) as control. The membranes were washed and incubated with AP-labeled secondary antibody (1:2000 dilutions) (Jackson, Lancaster, PA, USA) at RT for 2 h. The protein bands were then visualized by incubating with the developing solution (p-nitro blue tetrazolium chloride (NBT) and 5-bromo-4-chloro-3-indolylphosphate toluidine (BCIP)) at RT for 30 min. The relative densities of proteins were all determined by using ImageJ (NIH) v.1.33 u (Bethesda, MD, USA).

### 4.10. Mice Experiments

All animal experiments were conducted under protocols approved by the Animal Care and Use Committee of Ocean University of China (OUC-SMP-2023-09-14). All methods were performed in accordance with the animal ethics guidelines of the Chinese National Health and Medical Research Council (NHMRC). Four-week-old female BALB/c mice (average weight, 14.0 ± 2.0 g) were purchased from Jinan Pangyue laboratory animal breeding company (Jinan, China). Mice were inoculated intranasally with PR8 (500 PFU/mouse) diluted in 40 μL of 1 × PBS, and randomly divided into different experimental groups. Four hours after inoculation, mice received oral treatment of oseltamivir (10 mg/kg/day) (Roche, CH), baloxavir (10 mg/kg/day) (Shionogi, Osaka, Japan), MAE (5 or 10 mg/kg/day), or placebo, and the treatments were repeated once daily for the entire experiment. Mice were weighed and euthanized on Day 3 after inoculation by spinal dislocation method, and the lungs were removed and weighed. The lung specimens were homogenized in 1×PBS for determination of viral titers by NA titer assay. The left lobes of the lungs of three mice randomly selected from each group were fixed with 4% paraformaldehyde on Day 3. Then the tissues were dehydrated, paraffin embedded, sectioned, and stained with hematoxylin eosin (H&E).

In the survival experiments, 10 mice per group were intranasally infected with PR/8 virus (1000 PFU/mouse) at Day 0. Oseltamivir (10 mg/kg/day) or baloxavir (10 mg/kg/day) were used as positive control drugs. The drug administration was repeated once daily for seven days. Mice were monitored daily for weight loss and clinical signs. If a mouse lost body weight over 25% of its preinfection weight, it was defined as dead and humanely euthanized immediately; the rest of the mice were sacrificed at the end of experiment on 14 dpi.

### 4.11. Statistical Analysis

All data are representative of at least three independent experiments. Statistical significance was calculated by GraphPad Prism 8.0 (San Diego, CA, USA) using one-way analysis of variance (ANOVA), followed by post-hoc Tukey’s tests if F achieved statistical significance (*p* < 0.05) but there was no significant variance in homogeneity. The differences in mouse survival rates were compared using a log-rank (Mantel–Cox) test. Statistical significance was considered to be *p* < 0.05.

## 5. Conclusions

In conclusion, the novel compound MAE derived from marine fungus inhibits the infection of IAV both in vitro and in vivo and may block IAV replication through targeting the cellular Akt-mTOR-S6K pathway. Further studies on the antiviral effects of MAE against highly pathogenic IAV strains or clinical strains will be required to advance its drug development. The marine compound MAE has the potential to be developed into a novel anti-IAV agent for influenza therapy in the future.

## Data Availability

The data presented in this study are available on request from the corresponding author.
